# Observational descriptive study of cutaneous manifestations in patients from Mato Grosso with viral chronic hepatitis*

**DOI:** 10.1590/abd1806-4841.20153851

**Published:** 2015

**Authors:** Renato Roberto Liberato Rostey, Francisco José Dutra Souto

**Affiliations:** 1 Universidade Federal de Mato Grosso (UFMT) - Cuiabá (MT), Brazil; 2 Private clinic - Cáceres (MT), Brazil

**Keywords:** chronic Hepatitis B, chronic Hepatitis C, Lichen planus, Vitiligo

## Abstract

**BACKGROUND:**

Extrahepatic manifestations are seen in association with chronic infection by hepatitis B or C virus including cutaneous disorders. The frequency of these findings seems to vary among different places and reports. There is a lack of information about this issue in Brazil.

**OBJECTIVES:**

To estimate the prevalence of cutaneous findings affecting HBV or HCV carriers from a reference outpatient unit in Mato Grosso.

**METHODS:**

A cross-sectional observational study.

**RESULTS:**

108 patients were studied. 88.9% presented some cutaneous findings but must of them were nonrelated to chronic viral infection. Four patients had cutaneous or autoimmune syndromes that may be HBV or HCV related.

**CONCLUSION:**

In our study we found no statistical association between viral hepatitis and skin diseases.

## INTRODUCTION

### Viral hepatitis

More than 300,000 new cases of viral hepatitis were recorded between 1999 and 2010 in Brazil.^1^ It is a set of infectious diseases, often neglected by doctors and health services by the lack of symptoms, despite recent campaigns of the Ministry of Health of Brazil, aiming to educate the public and health professionals for early diagnosis and preventive measures.^2,3^ With increased search for the viral hepatitis, detection of chronic carriers has increased and hence a larger number of patients receiving drug therapy.^4^

Viral hepatitis are diseases caused by different viruses, which have in common the hepatocyte tropism.^5^ The distribution is universal, but with different levels of prevalence in each region. In Brazil, there are regional variations in the prevalence of each of the etiologic agents.^6^ Viral hepatitis, most often, are presented in an asymptomatic or oligosymptomatic form, and it is rarely diagnosed in the acute phase, gaining relevance by the large number of subjects with chronic forms and complications.^2,4^

### Viral hepatitis in the state of Mato Grosso

Between 1999 and 2009, were notified to the epidemiological surveillance 2,688 cases of hepatitis A, 2,809 cases of hepatitis B, 191 cases of hepatitis C, 17 cases of hepatitis delta and 14 cases of hepatitis E.^7^ However, a large proportion of diagnosed cases of hepatitis does not become identified as the exact etiology, being classified as "undetermined". That's because, despite recent improvements, public healthcare system is still precarious in attendance and in offering specific tests. In addition, these figures certainly represent underreporting, since only the number of patients with chronic hepatitis C treated between 2002 and 2008 by Sistema Único de Saúde (SUS) was 268.^8^ Another fact that shows the poor accuracy of this information is the notification of 14 cases of hepatitis E, a disease that does not have tests available in public healthcare system and that needs molecular biology tests to be confirmed. In fact, only one human case has been confirmed so far in the country.^9^

### Cutaneous manifestations in viral hepatitis

Although the virus is responsible for the viral hepatitis presents hepatocytes tropism, pathological manifestations of these infections can extrapolate the liver in some cases. Associations between HBV and polyarteritis nodosa, HBV and cryoglobulinemia, and between HCV and leukocytoclastic vasculitis, or even cryoglobulinemia with both infections, are classically known.^10^ In general, they occur in cases of chronic infection, but may also happen in acute infections, such as Gianotti-Crosti syndrome in hepatitis B and Guillain-Barre syndrome in hepatitis E.^2,10^

It is considered that these extrahepatic manifestations are probably derived from immunological phenomena triggered by intense or prolonged antigenic stimulation evoked by the virus, so it'd also occur with cutaneous manifestations.^2,10,11^

Cutaneous manifestations may also occur as adverse events of medications used in the treatment of viral hepatitis.^12-17^ It can happen due to abnormal liver function, and not properly to manifestation of viral infection, as in the case of porphyria cutanea tarda and hepatitis C.^18-20^ Also, some dermatological diseases appear to be cutaneous manifestations of acute and chronic viral infections, including hepatitis.^2,10,11,21-23^ We can find reports and citations about associations between dermatological diseases and viral hepatitis, but few try to explain how this interaction happens.^2,4,10,11,21,24-28^

The most frequently found associations were: acquired angioedema, papular acrodermatitis of childhood (Gianotti-Crosti), cryoglobulinemia, atopic dermatitis, neutrophilic dermatitis, erythema nodosum, erythema multiforme, necrolytic acral erythema, rash on sun-exposed area, lichen planus (in its different forms), skin necrosis, papular-purpuric gloves and socks, polyarteritis nodosa, rash, porphyria cutanea tarda, serum sickness-like syndrome, red fingers syndrome, urticaria and leukocytoclastic vasculitis.^2,4,10,29-38^

### Literature review

Matičiči (2007) suggests that lichen planus is an early marker of viral HCV infection, so if the infection is identified early, it can save lives.^39^

In Brazil, Freitas et al (2005), in a case-control study, found an increased prevalence of HCV in patients with lichen planus compared with the control group.^40^

In an Italian review of extrahepatic manifestations in chronic HCV infections, conducted by Galossi et al (2007), it was evidenced an association between porphyria cutanea tarda and oral lichen planus with HCV.^41^

Nagao and Sata (2012), in a case-control study of HCV infection and oral lichen planus (OLP) in Japan, showed the high frequency of OLP in patients with HCV, indicating that in all patients who may be diagnosed with OLP, the presence of HCV should be investigated.^42^

Raslan et al (2009), in a study of prevalence of skin diseases in 155 patients with HCV, found that 45.8% of them had some type of skin manifestation, and about half of them had just pruritus without any injury in the skin. It was also recorded that 5.2% had pigmented purpuric dermatitis, 3.9% had lichen planus, and 3.9% mouth ulcers. Leukocytoclastic vasculitis was found in only 2.6% of patients, and other skin disorders had lower prevalence. In the same study an ultrasound follow-up was conducted, relative to the size of the liver (increased, normal, cirrhotic). It was also evidenced one rash association with increased liver size and also to cirrhosis, with the largest relative risk in cirrhotic patients.^43^

The only study with a design that searched associations of systemic diseases related to oral lichen planus, performed by Tovaru et al (2013), in Romania, described a prevalence of 9.6% of patients positive for HCV. However, the most prevalent diseases (19%) were gallstone manifestations.^44^

Grossmann et al (2007), in a case-control study in Brazil, considered controversial the association between oral lichen planus and HCV. Among 50 patients with OLP, only one case presented positive result by serology to HCV. In the control group comprising 215 patients with chronic HCV, were found 20 patients (9.3%) with clinical diagnosis of lichen planus, two of them with associated cutaneous form.^45^

Bigby (2009), in the USA, conducted a meta-analysis of case-control studies and concluded that there is an association of HCV infection and lichen planus in regions such as East and Southeast Asia, Europe, Middle East and South America. Also, it was not verified association in: Southern Asia, Africa and North America.^46^

In a presentation of a clinical case, Paixão and Miot described a case of criobulinemia cutaneous vasculitis induced by HCV.^47^

There are also several reports of cases worldwide, which describe patients who develop OLP, LP and granuloma annulare after vaccination for HBV.^48,49^

The literature review on the association of infection by hepatotropic viruses and the occurrence of various skin diseases shows that there are still questions on this topic. Is there an association or not? Would these skin phenomena be extrahepatic manifestations of viral infection? Are they directly caused by these infectious agents or by immune processes induced by them? Is the simultaneous occurrence of these diseases just random, determined in some places due to a higher prevalence of one or another? Are skin changes induced by drugs used to control infections? Does genetic or geographical predisposition determine the differences between the studies?

As this issue has not been addressed in Midwest region of Brazil, a cross-sectional study was carried out to assess how the relation between these diseases occurs in this region.

### Case series

#### Type and place of study

We conducted an observational descriptive study of patients who were being followed or treated for viral hepatitis in the Infectious Diseases Service of Hospital Universitário Julio Müller (HUJM) at Universidade Federal de Mato Grosso (UFMT) in the city of Cuiabá.

#### Patients

They were eligible for the study all patients of the service described above, in clinical monitoring and/or treatment, with diagnosis of chronic viral hepatitis, in its different forms. Exclusion factor was co-infection with HIV, to avoid bias, as it is known to be a disease that competes with skin manifestations. To be included, all patients were informed of the research reasons, read and signed the informed consent form.

#### Studied variables

We collected demographic data (name, age, gender, address, occupation, skin type, weight and height). Classification of chronic viral hepatitis and all drugs used by patients, especially those specific agents for the treatment of hepatitis, were registered. Also, we recorded skin diseases prior to the diagnosis of viral hepatitis, reported by the patient, observed in the initial physical examination and those that occurred after the diagnosis of viral hepatitis.

#### Research procedure

All patients who were treated at the Infectious Diseases Service of HUJM-UFMT and diagnosed with viral hepatitis, and who didn't fit the exclusion criteria, were invited to participate in the study.

After consent, patients were submitted to an interview; they were asked about their diagnosis, diagnostic time, previous therapies and current treatment.

Patients answered a dermatological questionnaire, when they were asked if they've already presented some skin disease prior to diagnosis of viral hepatitis, if they still had the disease, if they presented some skin disease after diagnosis of viral hepatitis, and if they were presenting some skin disease at that time.

Then, all patients underwent a thorough dermatological clinical examination, which evaluated in a systematic manner all the skin, covering all body segments: scalp, oral mucosa, trunk, limbs, hands and feet, and its annexes (hair and nails).

Some patients, according to individual need, underwent revaluations for monitoring of dermatological diseases initially presented and treated.

Part of the information was obtained from medical records of patients selected for the study, such as details on the underlying disease (viral hepatitis), current medications and dose. Medical records were also researched for any dermatological history previously reported. A standardized form was used to record the initial and follow-up data of the patients.

#### Ethical aspects

The project was approved by the Ethics Committee of HUJM of UFMT, with the report Number 69287 of 08/08/2012 CEP-HUJM.

Participation in the study was completely voluntary. There was no refund or payment to patients who agreed to participate.

Patients who have been diagnosed with some skin diseases were treated for free by the researcher.

#### Data analysis procedures

All cutaneous events reported by patients, before or after diagnosis of viral hepatitis, were considered for statistical analysis, as well as any cutaneous events observed.

Data were tabulated and stored in a computerized database created with EpiData 3.0 software (Epidata Association, Odense Denmark 2002) and analyzed (EpiDataAnalysis) through intersection of variables and statistical analysis where appropriate.

## RESULTS

### Patient characteristics

We invited 111 patients to participate in the study. Three patients declined the invitation, so 108 patients comprised the sample. They were interviewed and examined between August 2012 and July 2013, in hepatology clinic of HUJM, and from these interviews we collected the data presented below.

Age ranged from 17 to 75 years, with a mean of 47.7 years. Regarding gender, 53.7% (58 patients) were male. Following the Fitzpatrick's scale as the color of skin, patients were divided into: one patient with skin type I; 18 patients with skin type II; 34 patients with skin type III; 35 patients with skin type IV; 16 patients with skin type V; and 4 patients with skin type VI. Of this total, 61 patients (56.5%) had HBV in chronic form and 47 patients (43.5%) had HCV in the chronic form. Patients with other forms of viral hepatitis were not found.

Regarding education, 7 (11.5%) of 61 patients with HBV were illiterate; 28 (45.9%) completed elementary school; 16 (26.2%) completed high school; and 10 had (16.4%) higher education. Among the 47 patients with HCV, one (2.2%) was illiterate; 19 (40.4%) completed elementary school; 19 (40.4%) completed high school; and 8 (17%) had higher education.

As for cutaneous manifestations of 61 patients with HBV, 17 (28%) had prior pathological history and 53 (87%) had some cutaneous manifestation during the examination. Among the 47 patients with HCV, 24 (51%) had positive history for skin diseases and 43 (91%) patients had some cutaneous manifestation during the examination.

There were no statistical differences by age or gender in the incidence of diseases found during physical examination in relation to statistical data obtained from literature.

### Medications in use

Seventy patients (64.8%) were taking medications. Of those using medications, these drugs were distributed in 62 different types, and 36 patients (33.3%) were using specific medications for the treatment of hepatitis. Some of these patients were taking more than one drug (Table 1). Among the 36 patients who were using specific medications for the treatment of hepatitis, 23 were in treatment for HBV and the remaining 13 were in treatment for HCV.

**Table 1:** Medications used in the treatment of viral hepatitis in patients with chronic hepatitis B or C assisted at hepatology clinic (Cuiabá 2012/2013)

Drugs:	n	%
Adefovir	1	1.9
Boceprevir	1	1.9
Entecavir	9	17.3
Interferon	4	7.7
Pegylated interferon	9	17.3
Lamivudine	4	7.7
Ribavirin	13	25.0
Telapravir	1	1.9
Tenofovir	10	19.2
**Total**	**52**	**100**

Other drugs in use at the time of interview belonged to groups of drugs for cardiac diseases, endocrine diseases, and digestive system disorders. Vitamins, antihistamines, antidepressants and others were also used (Chart 1).

**Chart 1:** Other medications used during the interview of patients with chronic hepatitis B or C at hepatology clinic (Cuiabá, 2012/2013)

Drugs:
Cardiological
digitalis
Angiotensin converting enzyme inhibitors
Angiotensin receptor 2
Class 3 antiarrhythmic
Statins
Thiazide diuretics
Calcium channel blockers
For endocrinopathies
Thyroxine
Corticosteroids
Insulin
Erythropoietin
Sulfonylureas
Biguanide
Thiazolidinediones
Bisphosphonates
Antidepressants and sedatives
Benzodiazepines
Serotonin reuptake inhibitors
Antipsychotics
Tricyclic derivatives
Tricyclic derivatives
For the digestive system
Proton pump inhibitors
Antispasmodics
Antiphysetics
Vitamins and trace elements
B complex
Folic acid
Ornithine
Ornithine
Antibiotics
Cephalosporins
Anti-inflammatory
Aspirin
Aspirin
Aspirin
Phosphodiesterase inhibitors (pentoxifylline)

As expected, many of the prescribed medications were commonly used pharmacological agents for liver disease, cirrhosis and its complications, such as diuretics, beta-adrenergic blockers, non-absorbable antibiotics and lactulose (Chart 2).

**Chart 2:** Medications commonly used to treat liver diseases, referred by patients with chronic hepatitis B or C from hepatology clinic at the time of interview (Cuiabá, 2012/2013)

**Drugs:**
Potassium-sparing diuretics
Spironolactone
Loop diuretics
Furosemide
Beta-adrenergic blockers
Propranolol
Metoprolol
Angiogenesis inhibitors (anticancer)
Sorafenib
Antibiotics
Fluoroquinolones (norfloxacin)
Aminoglycosides (neomycin)
Nonabsorbable disaccharides
Lactulose
"Hepatoprotector"
Silymarin

Fourteen patients had a history of previous use of medications for the treatment of hepatitis, but at the time they weren't using any specific medication. Among patients who had used specific medications for the treatment of viral hepatitis, the following data were found: two cases of skin rash associated with the use of ribavirin and pegylated interferon, a case of aracneiform hemangiomas and diffuse telangiectasias throughout the body (suggesting liver failure), and a case of lichen planus (prior to treatment).

### Characteristics of previous cutaneous manifestations at diagnosis of viral hepatitis

Of the 108 patients, 67 (62%) denied dermatological diseases prior to the diagnosis of viral hepatitis. Forty-eight different diagnoses were found among the 41 patients with positive medical history for dermatological diseases (Table 2).

**Table 2:** Patients with past medical history of cutaneous manifestations among those with viral hepatitis from HUJM's infectious diseases clinic (Cuiabá, 2012/2013)

Prior dermatological diseases	N	%
Leprosy	9	8.3
Pityriasis versicolor	7	6.5
Tinea (body or feet)	5	4.6
Acne	3	2.8
Enspecified dermatitis	3	2.8
Basal cell carcinoma	2	1.9
Scabiosis	2	1.9
Burn	2	1.9
Actinic keratosis	2	1.9
Venous ulcer	2	1.9
Lichen planus	1	0.9
Systemic lupus erythematosus	1	0.9
Other*	9	8.3
**Total**	**48**	**100%**

* A case of each of the following diseases: unspecified skin cancer, contact dermatitis, seborrheic dermatitis, erysipelas, herpes simplex, leishmaniasis, miliaria, congenital syphilis, unspecified ulcer

One patient had prior dermatological diseases that led to the diagnosis of viral hepatitis. This was a case of lichen planus, whose dermatologic diagnosis at the time led his doctor to suspect the possibility of concomitant viral hepatitis, which came to confirm hepatitis C.

### Dermatological characteristics at the time of the examination

Of the 108 patients, at the time of the physical examination, only 12 (11.1%) did not have any cutaneous manifestation. Ninety-six (88.9%) had at least one cutaneous manifestation (ranging from 1 to 3 per patient). In total, there we performed 142 diagnoses, and 67 were different diagnoses (Table 3).

**Table 3:** Prevalence of dermatologic diagnoses in patients with viral hepatitis from HUJM's infectious diseases clinic (Cuiabá 2012/2013)

Detected skin pathologies	N	%
Solar lentigines	14	12.9
Tinea (body, feet, nails)	13	12.0
Seborrheic dermatitis	9	8.3
Ochre dermatitis	7	6.5
Seborrheic keratoses	6	5.5
Melanocytic nevi	6	5.5
Poikiloderma of Civatte	5	4.6
Pityriasis versicolor	5	4.6
Hemangiomas (aracneiform, planus, ruby)	5	4.6
Acne	4	3.7
Other*	66	

* One to three cases of each of the following conditions: acrochordons, nail clubbing (hippocratic nails), basal cell carcinoma, scars, dermatofibroma, contact dermatitis by primary irritant, nummular dermatitis, acne papilomatous nigra, unspecified feet peeling eczematide hipocromiante, nodular elastosis with cysts and comedones (Favre Racouchot), solar elastosis, ecchymosis, solar erythema, acneiform eruption, neurotic excoriation, age senile ecchymosis, Raynaud's phenomenon, soft fibroma, folliculitis, leprosy, herpes simplex, herpes zoster, post inflammatory hyperchromia, sebaceous hiperplasisas, progressive macular hypomelanosis, vascular insufficiency, leukoderma gotata, leukonychia, lichen simplex, systemic lupus erythematosus, melasma, unspecified pruridermia, actinic keratoses, racial melanoniquea, miliaria, milios, pityriasis rosea, dilated pore of Winer, pseudoacanthosis nigricans, pseudofolliculitis of the beard, psoriasis, plantar keratoderma, keratosis pilaris, scalp seborrhea, telangiectasia (in thorax), stasis ulcer, wart vulgar and vitiligo.

Virtually all detected with dermatological diseases had well-defined etiology and not known relation to chronic viral infections of the liver. Some existing skin diseases, which could be a consequence of use of medications or immune response, will be discussed in the subsection below.

### Cutaneous manifestations potentially associated with chronic viral hepatitis, adverse events of medications or immune responses

Skin diseases that were found in this group and could potentially be related to viral hepatitis, to drugs used against hepatitis or to immunological manifestations of prolonged infections were: Raynaud's phenomenon, lichen planus, systemic lupus erythematosus, psoriasis and vitiligo (Table 4).

**Table 4:** Prevalence of dermatologic manifestations potentially associated with chronic viral hepatitis from HUJM's infectious diseases clinic (Cuiabá 2012/2013)

	N	%	Infection by
Raynaud's phenomenon	1	0.9	HCV
lichen planus	1	0.9	HCV
Systemic lupus erythematosus	1	0.9	HCV
Psoriasis	1	0.9	HCV
Vitiligo	1	0.9	HCV
**Total**	**5**	**4.5**	

Raynaud's phenomenon and systemic lupus erythematosus were present in the same patient, which dealt with those diseases for at least two years before the diagnosis of HCV. The patient was being treated with cyclosporine, hydroxychloroquine and prednisone. Regarding HCV, the patient was only being monitored, with no specific medication.

The case of psoriasis was referred to the hepatitis clinic due to positive serology for HBV, found during clinical investigation of the skin disease, which appeared 4 months before and was undiagnosed until then. It was confirmed that this was a case of chronic HBV, asymptomatic and inactive, being followed without a specific treatment.

The case of lichen planus was also diagnosed before confirmation of viral hepatitis during dermatologic consultation, whose assistant dermatologist decided to research the existence of viral hepatitis by knowing the possible association, and HCV was confirmed. Lichen planus disappeared after treatment for HCV with pegylated interferon and ribavirin. Currently the patient is being monitored, with no medications.

The case of vitiligo was the only one in which the emergence of skin disease was observed after diagnosis of hepatitis: it occurred exactly 24 weeks of the use of specific drugs for HCV - alpha pegylated interferon and ribavirin - as reported in the literature.

## DISCUSSION

Extrahepatic manifestations are described in chronic carriers of HBV and HCV hepatotropic viruses.^2,4,5,10-49^ Some of these conditions are expressed in the skin and its relation with viral hepatitis are explored by researchers around the world, such as the relation between lichen planus and HCV.^2,4,10,11,23-29,39-46^

It is important to know the relationship between some skin changes and chronic infection with these viruses, since the presentation of such skin diseases may indicate viral pathogens in cases where the virus was considered inactive. Also, skin disease identification knowingly associated with chronic viral hepatitis can draw the attention of dermatologists or physicians for the diagnosis of viral hepatitis, hitherto unsuspected.

On the other hand, cutaneous manifestations may also arise in chronic carriers of this virus, but triggered by drugs used to treat chronic hepatitis. Especially alpha-interferons that, being immunomodulators, can trigger immune-mediated manifestations or worsen autoimmune baseline diseases already manifested or about to manifest.

There is still a lot to learn in order to redeem doubts about the real magnitude of the association of certain skin diseases and chronic infections by hepatotropic virus. Some authors report that these manifestations are common among carriers of HBV or HCV, while others find no association among them, or even doubt them.^2,4,10,11,21,23,39,45,46,47^ Also, some authors go in the opposite direction, describing a high prevalence of viral markers in patients with skin diseases, such as the case of lichen planus.^28,41-44^ In some reviews and meta-analyzes, some authors suggest that there may be geographic variation between these associations, which can mean heterogeneity of services and studies, but may also indicate that genetic, cultural and environmental characteristics influence this relation.^45,46^

The vast majority of skin lesions found in this study are related to intense sun exposure, an expected condition because the study area is a region with high incidence of UVR. The presence of common diseases such as dermatophytosis and pityriasis versicolor are aggravated due to the tropical climate. Also, the presence of disesases of little relevance in the public or individual health point of view can be considered only findings among the numerous cutaneous manifestations described in dermatology.

Some dermatological manifestations, such as seborrheic dermatitis, Raynaud's phenomenon, herpes simplex and herpes zoster, lichen planus, psoriasis and vitiligo may have been, in some way, influenced by the immune status of the patient or triggered by viral antigen stimulation. Other manifestations, as scaling of hands and feet, skin rash, and pruridermia are usually related to adverse events of medications used to treat viral hepatitis.^13-17,22,29-31,33^

Anyhow, many of these conditions are common, such as seborrheic dermatitis, which affects 3% to 5% of the general population.^50^ Psoriasis has prevalence around 2% of world population.^50^ Lichen planus has prevalence below 1% of the general population.^50^ Raynaud's phenomenon has an estimated prevalence of 3-5% of the general population.^50^ Vitiligo has an incidence around 1-2% of the general population.^50^ The herpes virus has a serological prevalence that reaches about 90% of the adult population and its appearance is directly linked to the state of immunocompetence.^50^ In this study, these disorders weren't found more often than would be expected in the general population.

Although autoimmune diseases (psoriasis, vitiligo, lupus, Raynaud's phenomenon and lichen planus) were found in patients with hepatitis, the number of cases was not high (one of each), so it was not possible to suggest that some of these diseases were more common in these patients than in the general population. In Brazil, some Brazilian authors also didn't find skin changes at high frequency in patients with hepatitis B and C viruses that could be classified as manifestations of viral hepatitis. In this sense our data are consistent with national data, which cast doubts on the causal relationship between viral hepatitis and skin diseases.^45^

These findings, as already pointed out, join the findings of authors who believe there is no association between skin diseases and viral hepatitis, but only coincidences.^45,46,47^ The high prevalence of hepatitis C infection in patients with lichen planus is opposed to this theory.^24-28,39,40-44^

As we cannot be 100% sure about the correct date of contagion by viral hepatitis, because many patients are diagnosed late - many times because they are asymptomatic - or are diagnosed by chance, we suspect that these diseases may be related to the viral immune response.

Although this is consistent with results from other national and international authors, it is possible that the negative results presented are derived from the type II error, due to the reduced sample, the main limitation of this study. The period of time used to reach patients didn't allow including more cases. As a result, we do not know if with a larger sample, we would have had a chance to find more cases of skin diseases that have been associated over the years with chronic viral hepatitis.

Another factor that may have led to a low finding of cutaneous manifestations related to viral activity was the fact that 33.3% of patients with hepatitis B or C were being treated, and other 12.96% had undergone a prior specific treatment. With treatment by suppressing or inhibiting viral replication, some events may have slowed down and ceased to occur, not being perceived in the physical examination.

We think, given this situation, that it would be interesting to conduct new studies, not only in our region but also in other Brazilian regions, in order to clarify this interesting topic, involving skin changes induced by chronic infection by hepatotropic viruses.

## CONCLUSIONS

Few cases of cutaneous manifestations possibly related to hepatitis were detected.

We found a patient with Raynaud's phenomenon and systemic lupus erythematosus and another with psoriasis, apparently with no association between viral hepatitis and the diseases. A case of lichen planus served as a warning for the diagnosis of hepatitis C. Another case presented autoimmune disease (vitiligo) caused by the use of the treatment for hepatitis C (interferon-alpha).

Although 88.9% of the study population present some dermatologic manifestation, we cannot relate most of them with viral hepatitis as the most prevalent conditions found presented distinct and well-defined causes, unrelated to viral hepatitis or immunologically induced phenomena.

Frequency of cutaneous manifestations found in patients with viral hepatitis in Mato Grosso does not differ from expected frequencies for a population without these infections, in the studied sample.
